# Acknowledgement to Reviewers of *Healthcare* in 2019

**DOI:** 10.3390/healthcare8010019

**Published:** 2020-01-15

**Authors:** Healthcare Editorial Office

**Affiliations:** MDPI, St. Alban-Anlage 66, 4052 Basel, Switzerland

The editorial team greatly appreciates the reviewers who have dedicated their considerable time and expertise to the journal’s rigorous editorial process over the past 12 months, regardless of whether the papers are finally published or not. In 2019, a total of 170 papers were published in the journal, with a median time to first decision of 19 days and a median time from submission to publication of 43 days. The editors would like to express their sincere gratitude to the following reviewers for their generous contribution in 2019:
Abdullah Al-ZaghalAnnette BoazAdam B. EdwardsAnthony HerbertAdewuyi Ayodele AdeyinkaAnthony B. MillerAdrián CeccatoAntonia ArnaertAgnieszka JaworowskaAntonio LuqueAida BairamAntonio ClavennaAlbert FiguerasAntónio AbreuAlberto ModeneseAntonio J. Ramos-MorcilloAlejandro SanzAnurag SharmaAlessandro StefaniniApril D. DavisAlessandro AlaimoArifin SandhiAlessandro TonacciAristeidis H. KatsanosAlicia PeñalbaArmin DietzAlicja DomagalaAshish PathakAlonço VianaAsrat AmnieAlpo VuorioAssunção NogueiraAlvaro ToledoAstrid FinkÁlvaro Francisco Lopes De SousaAthina EconomouAmany HassanAurelio SessaAmbereen MehtaAwais FaridAmosy E M’KomaAyoub Al-JawaldehAmutha SelvamaniBachir BenarbaAmy ThrockmortonBahram SangelajiAna Belen Ortega AvilaBarbara Mabel DalyAndras LakosBenjamin AnsaAndreas RantalaBernhard RauchAndres R. SchneebergerBernhard ZelgerAndrew ChetwyndBhavneet WaliaÁngel Romero MartínezBill MuskAngela YangBlake HildrethAnjali ZarkarBo XiaAnn SvenssonBocong YuanAnna Sołtysik-PiorunkiewiczBonnie N. KaiserAnna M. SchotthoeferBram De BoerAnne KjemtrupBrendon YeeBrooke AllemangEgbert OosterwijkCarina Lundh HagelinElaine SturCarles ManeraElena RizaCarl-Olav StillerEleonora NuceraCarlos Romero MoralesElin A. BjörlingCarmine NovielloElizabeth Nappi CorrêaCaterina LeddaElsa PegadoCatherijne KnibbeEmma Forsén MantillaCésar Calvo LoboEmma BartfayChadwick C. CurtisErika Di ZazzoChafia Touil-BoukoffaEvgenia HalkiaCharis LiapiEwa LangeCharles MusselwhiteFátima RoqueChellakkan Selvanesan BlessonFederica SgangaChiara OppiFernando Fajardo-BullónChien-Feng LiFilipa F. ValeChih-Hao LinFilippa BonoChing-Fen JiangFilomena MoriscoChristian HirschFinn DiderichsenChristine Manlai KwanFor Yue TsoChristopher ToweFotios ChatzitheodoridisChristopher LambFrancesca GoriniClaire Ashton-JamesFrancesco PistelliClara Juandó-PratsFranco MuggiaClaudia PieperFulvio LausConnie LethinGabriela TopaConxita Mestres MirallesGaëtane CaesensCooky LimGang KouCorinna ReaGaynell M. SimpsonDan RomerGennaro CormioDan HorsfallGernot KronreifDaniel DibabaGianluca CataniaDaniel López-LópezGiorgina Barbara PiccoliDavid DíezGiulio FuscoDavid GeldmacherGiulio IlluminatiDavid Naranjo-HernándezGOTO YoshikazuDavid McDonaldGrace VincentDavid ZweikerGregg NelsonDavid LimGrzegorz MentelDavid HutchonGuang XuDenise TaylorGuangming ChengDesmond WhalenGuanshi ZhangDevayani BhaveGuido ErreygersDiana WoodsGuruswamy MaheshDiana LayneGustavo G. NascimentoDiana M. LayneGusztav BeltekiDirk Frans De KorneH.J. TangeDonald H. ShaffnerHae-chun RheeDong-Woo ChoHana MorrisseyE.A.M. BulderHan-Na KimEdith ClarosHans De LoofEdmundo Rosales-MayorHarishchandra DubeyEdward TolhurstHattie WrightHelena Fernández LópezJoel N. MaslowHelge StalsbergJohanna H. Van Der LeeHelmut FrohnhofenJohn HalperinHenrik AnderssonJohn F. NewmanHiroshi KurokiJohnny HollowayHiroto HondaJorge H. LeitãoHo LinJørgen Trankjær LauridsenHolly AhernJose MoranHolly B. Herberman MashJosé Garcia-AlonsoHolton AveryJOSE ANTONIO DE PAZHui XuJosé Enrique Torres VaamondeHui Ting ChngJosé Joaquín MiraHung-Chung HuangJosé M. Laínez-AguirreHyeongsu KimJosé R. VillarHyun Hee HeoJoseph CesarineIain ScottJoseph O. Falkinham IIIIan WalshJoukje SwinkelsIoannis IliasJulie WalabyekiIoannis E. TsolasJulie BalenIole VozzaJulie McCulloughIracema LeroiJun YasudaIsabel CastilloJung Tae(Steve) KimIsao OtsukaJürgen VormannIvo IlievJyotsna ShahJ. Christopher EhlenKaite YangJacek PyżalskiKaren SettyJames W. SonneKaren EislerJane MarkeKaren BishopJanet MattssonKarl BechterJaume Coll-FontKatarzyna SygitJavier Ortuño SierraKathleen GalvinJayoung HanKazuhiro P. IzawaJenil PatelKazumichi FujiokaJennifer FredianiKenneth LiegnerJennifer L. ScheidKenneth A. KnappJerald DumasKentaro OkunoJeroen Van GorkomKeun Ho RyuJeroen DikkenKeun-Yeong JeongJérôme BergerKezia GaitskellJessica McNeilKhalid El BairiJesus Prieto-LloretKimberly JamieJesús Adrián LópezKlaus KayserJhinuk ChatterjeeKoji NaruishiJia LiuKoji MatsuoJill Hines BennettKosuke MinagaJing FengKouichiro KawanoJinhong YangKriti PuriJitendra SinghKrzysztof LaudanskiJitka KlugarováKyaien ConnerJoachim StorsbergKyle B. WalshJoanne YaffeLadislav VolicerJoaquín Cayón De Las CuevasLaima SapezinskieneJoël WagnerLaiqun JinLaura NaegeleMaurits Van den NoortLaura SbaffiMeagen RosenthalLauretta LuckMegha SharmaLeonardo Juan Ramirez LopezMelanie WillsLewis CarpenterMelanie GentryLi-Fan LiuMelanie TurkLinda BaumannMelissa CaimanoLoredana IvanMellissa WithersLoredana SarmatiMichele PianaLorenzo DesideriMichele TotaroLorraine A. SheppardMihye ChoLouise UnderdahlMika SalmiLuana NosettiMinh Hoang NguyenLucia AnsaniMin-Seong HaLucia Ramirez-BaenaMinyon AventLukai LiuMitsushige SugimotoLynda SedikkiMiwa YamamotoLynne S. NemethMohamad ParnianpourMaddalena LettinoMohiuddin AhmedMaggie LongacreMónica Ríos-SilvaMakoto EndoMoreno BaruffiniManuel García-GoñiMunjae LeeManvi SharmaMunjae LeeMarcel HanischMuppala N. P. RajuMarcel Olde RikkertNagarjuna CheemarlaMarcia E. Herman-GiddensNasser ShararehMarcial Velasco GarridoNathanael OjongMarco DettoriNavneet RaiMaria LucaNeil ChadbornMaria OlenickNelda MierMaría Del Carmen Pérez-FuentesNeveen SaidMaria Francesca AlvisiNicholas B. DeFeliceMaria João De Almeida Coelho De SousaNicky BrittenMarianne MIddelveenNicola TurnerMaribel PinoNicola SilvestrisMarie B. CorkeryNicola GuessMarie-José H.E. GijsbertsNicola NanteMariel Colmán MartínezNina FlanaganMarijana BađunNnaemeka OdoMariusz DuplagaNoren HasmunMark DreherNOZAKI KosukeMarta TremoladaOrazio AielloMarta VasconcelosOrly SaridMartijn LambertPaiboon JungsuwadeeMary SharpPanagiotis PentarisMary W. MathisPaola LovaMaryam MalekigorjiPatrick D. KumavorMasafumi NozoePatrick Marius KogaMassimiliano F. PeanaPaul TulkensMassimo BracciPaul SchoenhagenMatteo MoroniPaul EganMatteo GulinoPaul E. LyonsMatthias KlumppPauline MarshPaweł WięchSara McNeillisPaweł ChruścielSara CampagnaPaweł PławiakSarah PriorPei Ling ChooSatoru YamadaPeter LeeScott R. SandersPeter MayneSema K. AydedePeter Bob PeerenboomSharanjot SainiPeyman ShokrollahiSharlene L. GomesPhilippa Howden-ChapmanShatadru ChakravartyPierre La RochelleShayu DeshpandePing ZouShervin AssariPingping HanShervin AssariPRAKASH KumarShervin MinaeeQihong DengShikha SainiQinyi WangShinichiro OchiQuan-Hoang VuongShino ObaQuentin Durand-MoreauShiqian Sherry GaoRahim MutluShuo ZhaoRajeev CherukupalliSimon EvansRamune JacobsenSimone GarzonRaoul FurlanoSimone AquinoRasa BarkauskienėSitalakshmi VenkatramanRefaat HegaziSoJung KimRenée J. BogschutzSou Hyun JangRicardo Martino AlbaStefan NilssonRichard BinghamStephanie WorrellRichard HainStergios BoussiosRichard Cawley MaddenSteven G. KovenRobert BransfieldSudheer RavuriRobert J. DonovanSudipta RakshitRobyn FivushSusan CorkRodolfo Rocha Vieira LeocádioSusan Madison-AntenucciRodrigo Poblete UmanzorSusanna McColleyRoger E. ThomasTakashi IsezakiRohail HassanTakatoshi HaraRosalie GreenbergTan Boon YeowRose MartiniTanisha JowseyRosemary HiscockTatiana ChristidesRoslyn LivingstoneTatsuya KondoRoy McConkeyTeresa LesiukRozeta SokouTetsuya HommaRuchi GeraThomas WanRüdiger KlapdorTim KilnerRuxandra Ioana Curea-PitoracTitto T IdiculaRyan RegoTobias KutznerRyan D. WilliamsonTomoki NakamuraSabino De GisiTsering StobdanSalome AdamU Rajendra AcharyaSalvador Cruz RambaudUmar ShafiqueSam TelfordUsama AwanSampath ParthasarathyUsha SambamoorthiSamuel Asumadu-SarkodieVaibhav JainSamuel N. BreitValentina GatteschiValerie McLaughlin CrabtreeXerxes Tesoro SeposoValerio PazienzaXuan HuiVenu VelagapudiYa-Wen LeeVett LloydYet Hong KhorVickie HughesYigang WeiVinay JasaniYinfeng ZhangVincenzo NataleYingjie SunVivian ValdmanisYongjae YooVivian KjellandYoshihiro IkuraVivienne TippettYubing ShiViviette AllenYuChun YaoWei-Hong ZhangYuelong JiWen LinYuhi FujimotoWendy A. StewartYulia SolovievaWen-Wei ChangYuliya MysyukWilf McSherryYung YauWilson AbreuYuriy BilanWim Van Den NoortgateYu-Tung HuangWolfgang LeisterZahra JalalWolfgang RascherZakir UddinWon LeeZhaowei KongWui-Chiang LeeZoe Kopsaftis

